# Rigid Polyurethane Foams Reinforced with POSS-Impregnated Sugar Beet Pulp Filler

**DOI:** 10.3390/ma13235493

**Published:** 2020-12-02

**Authors:** Anna Strąkowska, Sylwia Członka, Agnė Kairytė

**Affiliations:** 1 Institute of Polymer & Dye Technology, Lodz University of Technology, 90-924 Lodz, Poland; anna.strakowska@p.lodz.pl; 2 Laboratory of Thermal Insulating Materials and Acoustics, Institute of Building Materials, Faculty of Civil Engineering, Vilnius Gediminas Technical University, Linkmenu st. 28, LT-08217 Vilnius, Lithuania; agne.kairyte@vgtu.lt

**Keywords:** rigid polyurethane foams, sugar beet pulp, bio-filler, thermal conductivity, mechanical properties

## Abstract

Rigid polyurethane (PUR) foams were reinforced with sugar beet pulp (BP) impregnated with Aminopropylisobutyl-polyhedral oligomeric silsesquioxanes (APIB-POSS). BP filler was incorporated into PUR at different percentages—1, 2, and 5 wt.%. The impact of BP filler on morphology features, mechanical performances, and thermal stability of PUR was examined. The results revealed that the greatest improvement in physico-mechanical properties was observed at lower concentrations (1 and 2 wt.%) of BP filler. For example, when compared with neat PUR foams, the addition of 2 wt.% of BP resulted in the formation of PUR composite foams with increased compressive strength (~12%), greater flexural strength (~12%), and better impact strength (~6%). The results of thermogravimetric analysis (TGA) revealed that, due to the good thermal stability of POSS-impregnated BP filler, the reinforced PUR composite foams were characterized by better thermal stability—for example, by increasing the content of BP filler up to 5 wt.%, the mass residue measured at 600 °C increased from 29.0 to 31.9%. Moreover, the addition of each amount of filler resulted in the improvement of fire resistance of PUR composite foams, which was determined by measuring the value of heat peak release (pHRR), total heat release (THR), total smoke release (TSR), limiting oxygen index (LOI), and the amount of carbon monoxide (CO) and carbon dioxide (CO_2_) released during the combustion. The greatest improvement was observed for PUR composite foams with 2 wt.% of BP filler. The results presented in the current study indicate that the addition of a proper amount of POSS-impregnated BP filler may be an effective approach to the synthesis of PUR composites with improved physico-mechanical properties. Due to the outstanding properties of PUR composite foams reinforced with POSS-impregnated BP, such developed materials may be successfully used as thermal insulation materials in the building and construction industry.

## 1. Introduction

Rigid polyurethane (PUR) foams are porous materials, which are commonly used in many industries, including building engineering, automotive, construction, and furniture [1,2]. Currently, environmental awareness is increasing interest in sustainable development goals (SDGs) including sustainability and environmental protection. Because of this, the production of PUR composites with bio-based, organic fillers has attracted increased attention in the academic environment. Due to the low price, unlimited availability, and good physico-mechanical properties, such as low density and high stiffness, the cellulosic materials seem to be the most promising fillers for polymeric composites [3,4,5,6,7]. Many previous studies have shown, that the incorporation of cellulosic fillers may successfully improve the physico-mechanical characteristics of polymeric composites [8,9,10,11], including PUR foams [12,13]. For example, Silva et al. [14] produced rigid PUR foams reinforced with cellulose fiber residue from bleached eucalyptus pulp. The incorporation of cellulose fiber up to 16 wt.% has changed the properties of the composites, including the foam morphology and reducing cell size. PUR foams reinforced with 5, 10, and 15 wt.% of wood ash and fly ash fillers were prepared by Hejna et al. [15]. The introduction of both types of fillers caused some deterioration of the mechanical properties of PUR foams, such as compressive strength and flexural strength, but the obtained results were still considered satisfactory for most industrial applications. Moreover, the addition of fly/wood ash fillers improved the thermal stability of PUR foams reducing their thermal degradation. Interesting results were presented by Olszewski et al. [16] in the case of PUR composites modified with glass and sisal fibers–a significant improvement in mechanical properties of PUR composites was observed. Similar results were reported by Kurańska et al. [17]–the addition of 3–40 wt.% of basalt waste fillers resulted in the synthesis of PUR foams with improved mechanical characteristics. PUR foams reinforced with bamboo fibers (BF) with a particle size of 250–500 μm were produced by Li et al. [18]. When compared with neat PUR foams, the addition of BF increased the mechanical and flexural strength by ~47% and ~16%, respectively. Moreover, the results have shown that alkali treatment of BF results in better interaction between the BF surface and PUR matrix, leading to greater mechanical performances and better thermal stability of PUR foams. The impact of selected plant fillers, such as cinnamon extract, cocoa extract, and green coffee extract on the physico-mechanical properties of PUR foams was examined by Liszowska et al. [19]. It has been found that due to the incorporation of bio-based fillers, all series of PUR foams were more susceptible to biodegradation than the neat PUR foams. PUR foams reinforced with algal cellulose were produced by Jonjaroen et al. [20]. Due to the incorporation of algal cellulose, PUR foams were characterized by increased apparent density, greater stiffness and lower loss modulus when compared with neat PUR foams.

Sugar beet is one of the most commonly grown plants which is used in the sugar industry [21]. According to the Food and Agriculture Organization (FAO), sugar production from sugar beet is the second largest production in the world. The greatest amount of sugar beet is produced in Europe, Asia, and North America [21]. According to FAO, the production of sugar beet in Europe was 2772 million tonnes in 2016. The main problem with sugar production from sugar beets is the formation of a huge amount of waste-sugar beet pulp (BP), which consisting of polysaccharides (~70–80%), cellulose (~20%), hemicelluloses (~30%), and pectin (~25%) [22]. BP is mostly used as feed for animals, however, multiple different applications of BP have already been proposed–BP was used as an energy raw material, as a new source for bio-polyols for polyurethane synthesis as well as for paper manufacture [23]. For example, polyurethane foams were prepared from microwave liquefied sugar beet pulp (LSBP) and polymethylene polyphenyl isocyanate (PAPI) by Zheng et al. [24]. The effect of [NCO]/[OH] ratio on the mechanical, thermal, and microstructural properties of the LSBP–PU foams were studied. It has been shown, that as the [NCO]/[OH] ratio increased from 0.6 to 1.2, the LSBP-PU foams with the less regular structure deteriorated mechanical performances were produced. In another study, rigid polyurethane foams of significant renewable content (up to 50%) were produced using biomass biopolyols obtained via crude-glycerol mediated solvothermal liquefaction of sugar beet pulp and commercial diphenylmethane diisocyanate [25]. The produced foams exhibited higher apparent densities 43–160 kg m^−3^ and compressive strengths 34–254 kPa compared to tested commercial analogues. Biopolyol foams exhibited higher thermal stability and the non-flame retarded foams showed lower potential for fire spread due to lower pyrolysis gas combustion heat release rates and total released amounts of heat. Besides this, BP has not been used as a reinforcing filler for rigid polyurethane foams. Taking into account, the amount of BP, their availability, and production efficiency it seems logical and well-argued to use BP as a reinforcing filler in the PUR industry.

Previous studies have reported, that in the case of PUR foams reinforced with natural fillers the main concern is connected with the low thermal stability of bio-based organic fillers, which consequently, deteriorates the thermal stability of final products [26]. The incompatibility between the hydrophilic surface of cellulose fillers and the hydrophobic structure of PUR results in interfacial separation, leading to deteriorated performances of PUR foams [27]. Because of this, the modification of the filler surface seems to be a crucial step before the incorporation of organic fillers into the PUR matrix. Up to now, various attempts have been reported to improve the compatibility of the fillers with the polymeric matrix [28,29,30,31,32]. Several authors have reported different surface modifications for organic fillers, which involve chemical modifications, such as acetylation [33], alkalization [34], benzoylation [35], grafting [35], and silane treatment [36]. The results have shown, that the surface modification of organic fillers results in the production of polymeric composites with improved thermal and mechanical characteristics.

Many previous works have studied the impact of natural fillers on the mechanical and thermal characteristics of polymeric composites, however, no studies have been devoted to the examination of the polyurethane composite foams reinforced with beet pulp (BP) filler physically impregnated with thermally stable inorganic compounds, such as Polyhedral Oligomeric Silsesquioxanes (POSSs). POSS compounds are organic-inorganic compounds. The chemical structure of POSS includes a silica core and oxygen atoms at the edges of the molecule [37,38]. Our previous studies have shown that the incorporation of POSS molecules into PUR foams may improve the thermal stability and degradation behavior of PUR foams, as well as their mechanical performances [39,40,41,42]. Keeping in view the advantageous properties of beet pulp filler and POSS compounds, it seems logical to use POSS-impregnated BP filler as a reinforcing, hybrid filler for new bio-based polyurethane composite foams. The preparation of novel materials from POSS-impregnated BP filler may successfully improve the mechanical and thermal properties of the polyurethane materials. Therefore, in this study, rigid PUR foams were reinforced with 1, 2, and 5 wt.% of BP filler impregnated with Aminopropylisobutyl-POSS (APIB-POSS). The impact of POSS-impregnated BP filler on the morphology and physico-mechanical performances of PUR composite foams was examined.

## 2. Materials and Methods

### 2.1. Materials

Polyether polyol (commercial name: Stapanpol PS-2352, Northfield, MN, USA), polymeric diphenylmethane diisocyanate (commercial name: Purocyn B, Purinova Sp. z o.o., Bydgoszcz, Poland), potassium octoate (commercial name: Kosmos 75, Evonik Industries AG., Essen, Germany), and potassium acetate (commercial name: Kosmos 33 (Evonik Industries AG., Essen, Germany) were used as catalysts. Tegostab B8513 (Evonik Industries AG., Essen, Germany) was used as a silicone-based surfactant. A mixture of pentane and cyclopentane (50:50 *v*/*v*%) was used as a blowing agent. Aminopropylisobutyl-POSS (APIB-POSS) was purchased from Hybrid Plastics Inc (Hattiesburg, MS, USA). Sodium hydroxide (pellets, anhydrous), was purchased from Sigma-Aldrich Corporation (Saint Louis, MO, USA). Sugar beet pulp was obtained from a local company (Lodz, Poland).

### 2.2. Filler Preparation and Synthesis of PUR Composite Foams

**Figure 1:**
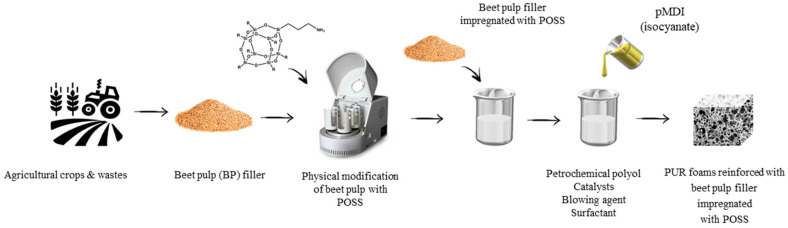
Schematic procedure of the synthesis of PUR composite foams.

PUR composite foams were synthesized according to the procedure described previously [43]. The selected amount of beet pulp (BP) filler was added to the polyol system. Before the addition, the BP filler was alkali-treated with 5 wt.% solution of sodium hydroxide solution using the method presented in [26]. After the alkali treatment, such prepared BP filler was mixed with Aminopropylisobutyl-POSS (APIB-POSS) (1:1 *w*/*w*) using a planetary ball mill for 1 h (3000 rpm). Then, the calculated amount of other ingredients, such as a surfactant, catalyst, and blowing agent was added to the polyol system and the mixture was intensively mixed by mechanical stirrer at 1000 rpm for 60 s. After the complete dispersion of BP filler, a calculated amount of isocyanate was added to the mixture and mixed at 2000 rpm for 30 s. Such prepared composite foams were left to expand freely at room temperature. To provide complete curing, PUR composite foams were conditioned at room temperature for 48 h. The formulations of PUR composite foams containing BP filler are presented in Table 1. The schematic procedure of PUR composite foams preparation is presented in Figure 1. Different concentrations of BP fillers were used to determine the impact of filler content on the further properties of PUR composite foams. The reference PUR foams were prepared without the addition of BP filler.

### 2.3. Sample Characterization

The dynamic viscosity was determined according to ISO 2555 [44] using Viscometer DVII+ (Viscometer DVII+, Brookfield, Berlin, Germany). The morphology of PUR composite foams was determined using a scanning electron microscope (SEM) (JEOL JSM 5500 LV, JEOL Ltd., Peabody, MA, USA). The cell sizes of PUR composite foams was determined by ImageJ software (Java 1.8.0, Media Cybernetics Inc., Rockville, MD, USA). The apparent density of PUR composite foams was determined in accordance with ISO 845 [45]. The number of closed-cells was evaluated according to ISO 4590 [46]. Thermal conductivity (λ) of PUR composite foams was measured using LaserComp 50 (TA Instruments Inc., New Castle, DE, USA). The mechanical properties (compressive strength, flexural strength) of PUR composite foams were performed using Zwick Z100 Testing Machine (Zwick/Roell Group, Ulm, Germany) according to ISO 844 [47] and ISO 178 [48]. The impact strength was determined using the Charpy Impact Strength Test Machine according to ISO 180 [49]. The thermogravimetric analysis (TGA) test was performed in the function of temperature (0–600 °C) using the STA 449 F1 Jupiter Analyzer (Netzsch Group, Selb, Germany). Surface hydrophobicity of PUR composite foams was measured using contact angle goniometer OEC-15EC (DataPhysics Instruments GmbH, Filderstadt, Germany). A cone calorimeter test was performed using the cone calorimeter apparatus according to ISO 5660 [50].

## 3. Results and Discussion

### 3.1. Filler Characterization

**Figure 2:**
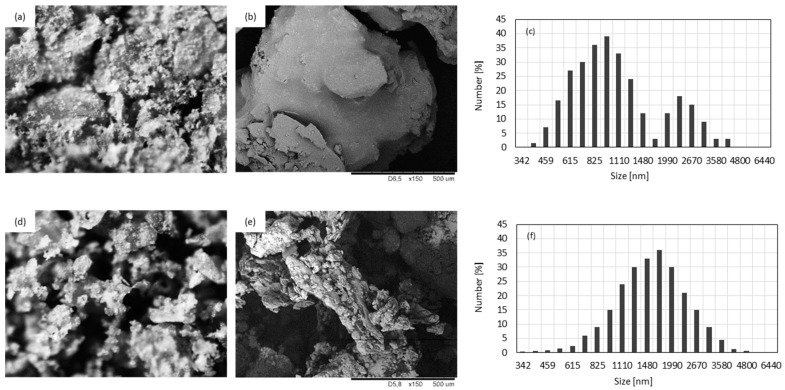
External surface and particle size of neat BP filler (**a**–**c**) and BP filler impregnated with POSS (**d**–**f**).

An external topography of neat BP and BP modified with POSS is presented in Figure 2. Comparing SEM images, it is clear that the physical modification of BP filler with POSS compounds affected the overall structure of the filler. After the modification, the external surface of BP filler became more rough and non-uniform. The crystals of POSS particles are visible on the surface of the BP filler. The size of neat BP filler is in the range of 400–800 nm, however, the filler particles tended to agglomerate and some bigger clusters with an average diameter of ~3 µm are presented. After the modification, the size of filler particles increased to ~2 µm due to the impregnation of BP filler’s surface with POSS molecules. When compared with neat BP filler, the size distribution of modified BP filler is more uniform, which indicates that the physical modification with POSS prevented the filler particles from agglomerating. Moreover, the addition of modified BP filler affected the viscosity of PUR systems (Table 2)—when compared with neat PUR system, the viscosity increases from 800 to 1550 mPa·s, for PUR system with 1, 2, and 5 wt.% of BP filler, respectively.

### 3.2. Foaming Behavior of PUR Systems

The viscosity of PUR systems has a great impact on the foaming behavior of porous materials. The foaming behavior of PUR foams was examined by measuring start, free-rise, and tack-free times. According to the results given in Table 2, the processing times tended to increase with the addition of BP filler. When compared with neat PUR systems, the addition of 1, 2, and 5 wt.% of BP filler increased the start time by ~15, ~25, and ~41%, while the free-rise time increased by ~9, ~14, and ~29%, respectively. The main reason for extended times may be attributed to the increased viscosity of PUR systems with BP filler, which limits the proper expansion of the cells and consequently extends the processing times of PUR systems. This effect is more noticeable in the case of PUR systems with 5 wt.% of BP filler. In general, the higher the BP filler content, the greater the viscosity of the PUR systems and the longer their processing times. On the other hand, the addition of BP filler may decrease the reactivity of PUR systems, due to the incorporation of the additional groups of BP filler, which are able to react with highly reactive isocyanate groups. Due to this, a greater amount of isocyanate is consumed, which affects the proper stoichiometry of the PUR synthesis and reduces the amount of carbon dioxide (CO_2_) produced. A reduced amount of blowing agent results in limited expansion of the cells, extending the processing times of PUR synthesis. Similar results have also been found in previous works [51,52].

### 3.3. Cellular Structure of PUR Composite Foams

Physical and mechanical properties of porous materials are affected by their cellular structure; therefore, the examination of foams’ structure is vital. In order to determine the microscopic morphology of PUR composite foams with BP filler, SEM analysis was examined.

**Figure 3:**
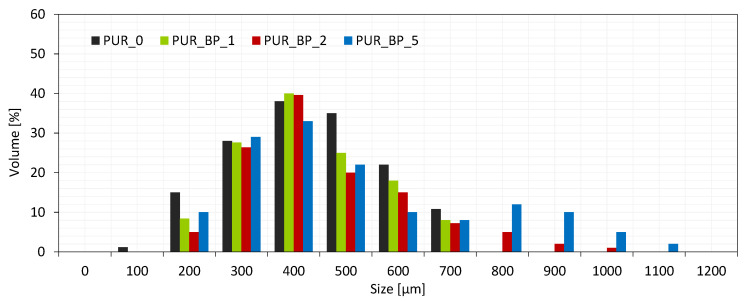
The cell size distribution of PUR composite foams with BP filler.

According to the results presented in Table 3, the average cell diameter of neat PUR foams is 492 µm. The cell diameter increases from 450 to 530 µm with the increase in BP content from 1 to 5 wt.%. As presented in Figure 3, the cell size distribution of PUR composite foams containing 1 and 2 wt.% is uniform and it is distributed between 200 and 700 µm. With the addition of 5 wt.% of BP filler, the cell size distribution becomes wider and some bigger cells are visible in the PUR structure. It can be concluded that lower content of BP filler can be mixed with polyurethane system homogenously, leading to the formation of a PUR structure with uniform cell size distribution. Moreover, the incorporation of BP fillers provides additional nucleation sites which result in the formation of a higher number of smaller cells when compared with neat PUR foams. The addition of BP fillers increases the viscosity of PUR systems, reducing the increase in cell size. Increasing the content of BP filler up to 5 wt.% deteriorates the structure of PUR composite foams. This may be connected with the agglomeration of BP filler after a certain content due to the high viscosity of the PUR system. The agglomeration of filler particles results in inhomogeneous distribution and the formation of uneven foam structures with bigger cells.

**Figure 4:**
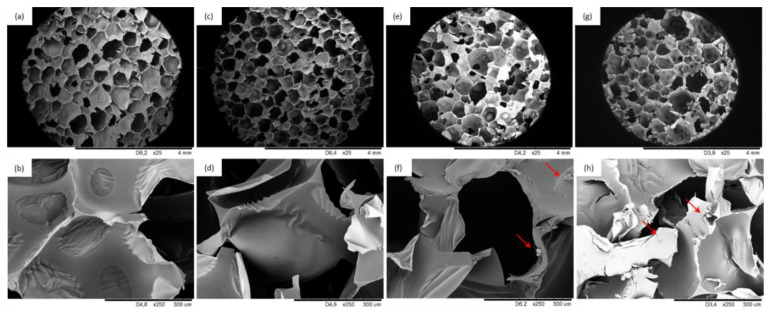
Cellular morphologies at different magnifications of (**a**,**b**) PUR_0, (**c**,**d**) PUR_BP_1, (**e**,**f**) PUR_BP_2, (**g**,**h**) PUR_BP_5.

Figure 4 presents the overall structure of PUR composite foams with various BP filler content. Generally speaking, by increasing the content of BP filler, the overall structure of PUR composite foams becomes less uniform with a higher number of cracked cells. This may be ascribed to the following reasons: BP filler particles act as nucleation sites for the gas phase, affecting the rheology around the growing air bubbles and changing the nucleation character from homogenous to heterogeneous [53]. This results in the creation of a higher number of finer cells, which tend to collapse, resulting in a less homogeneous structure of PUR composite foams. Moreover, the poor interphase compatibility between filler surface and PUR matrix promotes earlier collapsing of the cells, leading to the formation of a more defective structure of PUR composite foams [54]. This effect is most prominent in the case of PUR composite foams with higher loading of BP filler, due to the significant agglomeration of the filler, which promotes the rupturing of the cells and creating a weakened PUR structure.

### 3.4. Apparent Density of PUR Composite Foams

**Figure 5:**
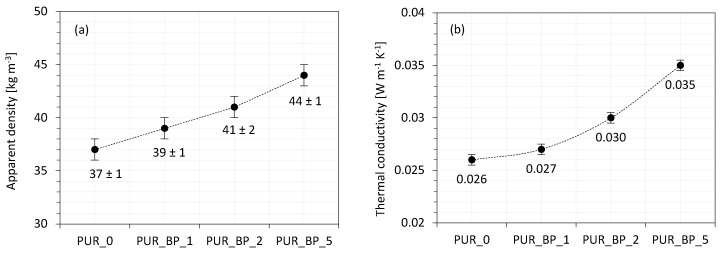
The results of (**a**) apparent density and (**b**) thermal conductivity of PUR composite foams.

The results of the apparent density of PUR composite foams with different BP content are presented in Figure 5a. The density of PUR composite foams increased from 37 kg m^−3^ to 40, 41, and 44 kg m^−3^ after the addition of 1, 2, and 5 wt.% of BP filler, respectively, due to the following reasons. Firstly, the density of modified PUR composite foams is enhanced due to the incorporation of BP filler which possesses a higher density than neat PUR foams. Therefore, the density of the modified PUR composite foams increased with the increasing content of BP filler. Furthermore, the addition of BP filler increases the viscosity of the PUR systems and reduces the expansion of the cells. Thus, the density of PUR composite foams increased with the increase in the BP filler content.

### 3.5. Thermal Conductivity of PUR Composite Foams

The cellular structure and apparent density of PUR composite foams affect their insulating properties. The value of thermal conductivity (λ) of neat PUR_0 is 0.025 Wm^−1^ K^−1^. The addition of 1 and 2 wt.% of BP filler had no effect on λ, however, the addition of 5 wt.% of BP filler increased the value of λ to 0.035 Wm^−1^ K^−1^ (Figure 5b). In general, thermal conductivity is determined as a combination of λ_gas_, λ_solid_, and λ_convection_. Due to the incorporation of solid particles of BP filler, which are built in the PUR matrix, the value of λ_solid_ increases. This effect is most prominent in the case of PUR composite foams with 5 wt.% of BP filler. As discussed previously, BP filler particles tend to agglomerate, resulting in the formation of a greater number of open cells. This, in turn, increases the value of λ_gas_, worsening the insulating properties of PUR composite foams. Similar behavior was observed in previous works, concerning the PUR composite foams modified with the addition of selected organic and/or inorganic fillers. For example, an increased value of λ was reported for PUR foams with 3–40 wt.% of basalt waste filler by Kurańska et. al. [17]. It has been shown that besides the well-developed, closed-cell structure of modified PUR foams, the insulating properties are deteriorated, however, following industrial standards, they are still considered at an acceptable level.

### 3.6. Contact Angle and Water Uptake of PUR Composite Foams

Water uptake depends on morphological features of PUR foams (e.g., the content of open/closed-cells) as well as on the hydrophobic nature of the filler [55]. According to the results presented in Table 3, the addition of BP filler affects the water uptake of PUR composite foams. When compared with neat PUR_0, the addition of 1 and 2 wt.% of BP filler decreased the water uptake by ~9 and ~7%, while for PUR composite foams with 5 wt.% of BP filler, the value increased by ~10%. As presented in SEM images (Figure 4), the addition of 5 wt.% results in the opening of cells which are able to store a greater amount of water. Reduced water uptake of PUR composite foams with 1 and 2 wt.% of BP filler may be connected with a greater number of closed-cells that are not able to accommodate water. Furthermore, this effect may be also enhanced by the hydrophobic character of incorporated filler, which was also confirmed by the results of the contact angle (Table 3). However, the impact of the cellular structure and the content of open/closed-cells seems to be a more dominant factor, which determines water uptake ability.

### 3.7. Mechanical Performances of PUR Composite Foams

**Figure 6:**
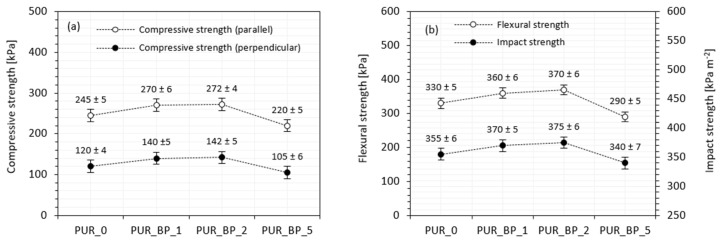
The results of (**a**) compressive strength and (**b**) flexural/impact strength of PUR composite foams.

Mechanical performances of porous materials, including PUR foams, are dependent on several factors, such as cellular structure (e.g., shape and size of cells) and density of PUR foams [56]. Furthermore, the mechanical performances of PUR composite foams are influenced by the homogenous dispersion of the fillers in the PUR system and the interphase compatibility between the filler surface and the PUR matrix. Figure 6a presents the compressive strength of PUR composite foams containing various contents of BP filler. The compressive strength of PUR composite foams increases initially with the addition of 1 and 2 wt.% of BP filler, and then decreases slightly with the further increase in BP filler up to 5 wt.%. When compared with neat PUR foams, the greatest compressive strength is observed on the addition of 2 wt.% of BP filler—the value of compressive strength (measured parallel) is 272 kPa, which is ~12% higher than that of the neat PUR foams. A similar tendency is observed in the case of compressive strength measured perpendicular to the direction of foam growth, but the compressive strength values are much lower due to the anisotropy of the foam cells [57]. Similarly, the greatest improvement is observed for PUR composite foams with the addition of 2 wt.% BP filler—compressive strength (measured perpendicularly) increases by ~18%. An increase in the formation of a greater number of smaller cells, contributes to a superior mechanical behavior of PUR composite foams; the applied load is encountered by a greater number of cell walls per unit area. This indicates that the addition of 1 and 2 wt.% of BP filler has a reinforcing effect and increases the mechanical performance of PUR composite foams. The obtained results meet the industrial requirements for constructive materials (apparent density > 35 kg m^−3^, σ_10%_ > 200 kPa) [58]. The addition of BP filler at an optimal level and the appropriate distribution of filler particles in the cell struts strengthened the foam structure, increasing the compressive strength. Increasing the BP filler up to 5 wt.% causes a BP filler agglomeration, which in turn, results in filler–filler interaction, deteriorating the mechanical performances of PUR composite foams [59]. As reported in SEM images (Figure 4), at higher content of BP filler the overall structure of the PUR composite foams becomes distorted and some voids are visible, deteriorating their mechanical properties. To eliminate the effect of density on mechanical performances of PUR composite foams, the specific compressive strength was calculated as a ratio of compressive strength to density. The specific strength of neat PUR foams is 6.5 MPa/kg/m^3^ and it increases insignificantly to 6.9 and 6.6 MPa/kg/m^3^ for PUR_BP_1 and PUR_BP_2, respectively, and then decreases to 5.1 MPa/kg/m^3^ for PUR_BP_5.

According to the results presented in Figure 6b, the addition of 1 and 2 wt.% of BP filler results in an improvement in flexural and impact strength, although an insignificant deterioration in properties is observed on the addition of 5 wt.% of BP filler. The greatest improvement in flexural and impact strength is observed on the addition of 2 wt.% of the filler—the value of flexural and impact strength increases by ~12 and ~6%, respectively. This result may be connected with the fact that filler particles act as additional stress points for the local stress concentration from which the cracking of the sample begins [60,61]. As the concentration of BP filler increases, the effect is more prominent, leading to the deterioration of the abovementioned properties.

### 3.8. Thermal Stability of PUR Composite Foams

**Figure 7:**
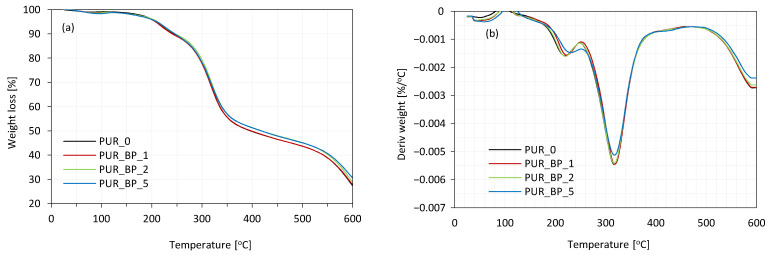
(**a**) TGA and (**b**) DTG results obtained for PUR composite foams.

Thermogravimetric (TGA) and derivative thermogravimetry (DTG) analysis of BP fillers and PUR composite foams are presented in Figure 7a,b, respectively. The resulting parameters are summarized in Table 4.

PUR composite foams show three stages of weight loss. The first stage (T_max1_) occurs around 200–250 °C and refers to the release of volatile compounds of BP filler [62]. When compared with neat PUR_0, T_max1_ increases from 220 °C to 224 and 226 °C for the composite foams with 1 and 2 wt.% of BP filler, respectively, and then decreases to 217 °C for the composite foams with 5 wt.% of BP filler, due to the heterogeneity of the PUR structure caused by filler agglomeration. T_max2_ occurs in the range of 300–350 °C and refers to the degradation of soft segments of PUR and thermolysis of the residues of organic BP filler [57,63]. The value of T_max2_ of neat PUR foams is 309 °C. Due to the addition of 1 and 2 wt.% of BP filler, the value of T_max2_ increases slightly to 325 and 320 °C, respectively. Such improvement may be due to the fact that fuller particles act as a thermal barrier, which prevents rapid heat transfer and reduces further degradation of PUR composite foams. Such an effect is further enhanced by a greater crosslinking of PUR, due to the reaction between functional groups of BP filler (i.e., hydroxyl groups) and isocyanate groups. The value of T_max2_ decreases slightly for PUR foams with 5 wt.% of BP filler, due to the more open-cell structure. T_max3_ occurs in the range of 500–600 °C and corresponds to the thermal degradation of cellulosic derivatives of BP filler [64,65]. Due to the presence of lignocellulose filler, the value of T_max3_ slightly increases at the addition of BP filler. Moreover, with increasing the content of BP filler, the mass residue (at 600 °C) increases from 29.0 (for neat PUR_0) to 29.1, 30.7, and 31.9% for PUR composite foams with 1, 2, and 5 wt.% of BP filler, respectively. Due to the great thermal stability of PUR composite foams, such developed materials may be successfully used as thermal insulation materials in the building and construction industry.

### 3.9. Cone Calorimeter Test

**Figure 8:**
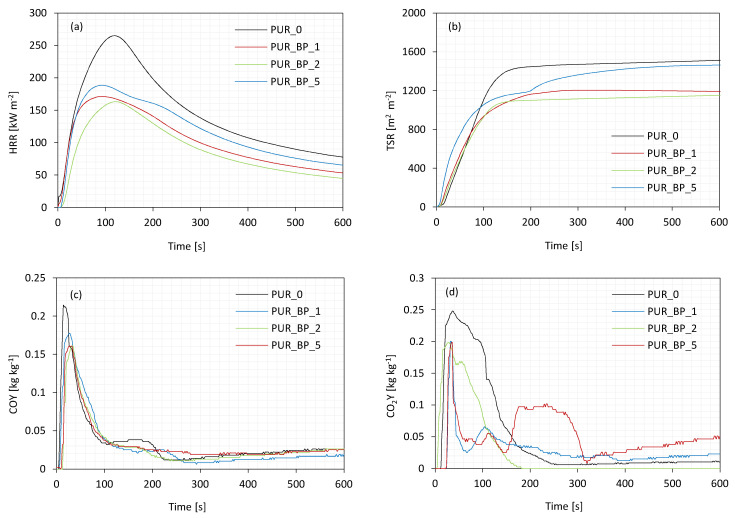
The results of the cone calorimeter test—(**a**) heat peak release (pHRR), (**b**) total smoke release (TSR), (**c**) CO release, and (**d**) CO_2_ release.

The results of fire behavior of PUR composite foams are presented in Table 5 and Figure 8.

**Figure 9:**
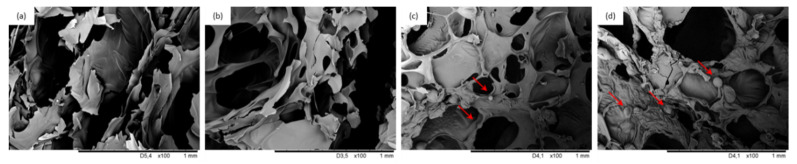
SEM images of char residues after the cone calorimeter test of (**a**) PUR_0, (**b**) PUR_BP_1, (**c**) PUR_BP_2, (**d**) PUR_BP_5.

Compared to neat PUR_0, the addition of BP filler has no effect on the ignition time (IT)—in all cases, the value of IT oscillates between 4 and 5 s. The intensity of the flame was determined by measuring the value of heat peak release (pHRR). As presented in Figure 8a, all series of PUR composite foams exhibited one peak of HRR, which refers to the release of low molecular weight compounds of PUR foams, such as isocyanate, amines, or olefins. When compared with neat PUR_0, the pHRR value decreases from 260 kW m^−2^ to 170 and 155 kW m^−2^ for PUR_BP_1 and PUR_BP_2 and then increases to 190 kW m^−2^ on the incorporation of 5 wt.% of BP filler. Such improvement in the fire resistance of PUR composite foams may be connected with the creation of a continuous, distended char layer on the surface of PUR foams with the addition of BP filler, which acts as a physical barrier and effectively limits the mass and heat transfer [66]. Moreover, the BP filler degrades endothermically and during the decomposition releases non-combustible products, which decreases the rate of heat release. Among all series of PUR composite foams, the greatest improvement is observed for PUR composite foams with 1 and 2 wt.% of BP filler—the value of pHRR decreases by ~35 and ~40%, respectively. In the case of PUR composite foams with 5 wt.% of BP filler, the value of pHRR decreases slightly by ~27%, due to their more open-cell structure when compared with PUR with lower content of the filler. As presented in Figure 8b, the incorporation of each amount of BP filler results in a lower value of total smoke release (TSR). When compared with neat PUR_0, the value of TSR decreases by ~20, ~27, and ~3%, for PUR composite foams with 1, 2, and 5 wt.% of BP filler. This indicates that incorporation of BP filler prevents the heat transfer and protects the PUR structure from further combustion [67]. Moreover, the incorporation of BP fillers decreases the value of total heat release (THR). Compared to neat PUR_0, the addition of 1 and 2 wt.% of BP filler decreases the value of THR from 21.5 MJ m^−2^ (for PUR_0) to 20.5 and 20.9 MJ m^−2^, respectively. No significant difference is observed for PUR composite foams with 5 wt.% of BP—the value of THR decreases slightly to 21.2 MJ m^−2^. As presented in Figure 8c,d, the incorporation of BP filler decreases the ratio of carbon monoxide (CO) to carbon dioxide (CO_2_), which refers to the foam toxicity. In general, a higher value of the ratio (CO/CO_2_) indicates incomplete combustion of PUR composite foams and a greater amount of toxic smoke. The addition of BP filler reduces the value of the ratio, which means that the release of toxic smoke during the PUR foams combustion is reduced. The outer structure of the char residue of PUR composite foams is presented in Figure 9. It can be observed that the addition of 1 wt.% of BP filler has no effect on the morphology of the char residue layer. The difference is observed in the case of PUR composite foams with the addition of 2 and 5 wt.% of BP—the outer surface becomes more dense and the spherical carbon residues are visible in the structure. It can be concluded that BF particles can act as a flame barrier limiting the release of combustible gases.

The improvement in flame resistance of the PUR composite foams with BP filler has been confirmed by the results of limiting oxygen index (LOI). As presented in Table 5, the addition of BP fillers increases the value of LOI of PUR foams. The greatest improvement was observed for PUR foams with 1 and 2 wt.%—the value of LOI increased from 20.2% (for neat PUR_0) to 20.9 and 21.2%, respectively. A less noticeable improvement was observed for PUR foams with 5 wt.% of BP filler—the value of LOI increased to 20.5%. Similar results have been reported in previous works as well.

## 4. Conclusions

Rigid polyurethane (PUR) foams were reinforced with sugar beet pulp (BP) impregnated with Aminopropylisobutyl-POSS. BP filler was incorporated into PUR at different percentages—1, 2, and 5 wt.%. The influence of BP filler on morphological features, mechanical properties, and thermal stability of PUR composite foams was investigated. The results showed that the greatest improvement in physico-mechanical properties was observed at a lower concentration of BP filler, such as 1 and 2 wt.%. For example, when compared with neat PUR foams, the addition of 2 wt.% of BP resulted in the formation of PUR composite foams with increased compressive strength (~12%), greater flexural strength (~12%), and better impact strength (~6%). Due to the good thermal stability of POSS-impregnated BP filler, the reinforced PUR composite foams were characterized by better thermal stability. Moreover, the addition of each amount of filler resulted in improvements in the fire resistance of PUR foams—the greatest improvement was observed for PUR composite foams with 2 wt.% of BP filler. The results presented in the current study indicate that the addition of a proper amount of POSS-impregnated BP filler may be an effective approach to the synthesis of PUR composite foams with improved physico-mechanical properties.

## Figures and Tables

**Table 1:** Formula of neat polyurethane (PUR) foams and PUR composite foams.

Component	PUR_0	PUR_BP_1	PUR_BP_2	PUR_BP_5
	Parts by Weight (wt.%)			
STEPANPOL PS-2352	100	100	100	100
PUROCYN B	160	160	160	160
Kosmos 75	6	6	6	6
Kosmos 33	0.8	0.8	0.8	0.8
Tegostab B8513	2.5	2.5	2.5	2.5
Water	0.5	0.5	0.5	0.5
Pentane/cyclopentane	11	11	11	11
Beet pulp impregnated with polyhedral oligomeric silsesquioxanes (POSS)	0	1	2	5

**Table 2:** Dynamic viscosity and processing times of PUR systems with BP filler.

Sample	Dynamic Viscosity *η* (mPa·s)	Processing Times (s)		
		Cream Time	Free-Rise Time	Tack-Free Time
PUR_0	800 ± 9	39 ± 3	282 ± 9	360 ± 10
PUR_BP_1	980 ± 10	45 ± 2	308 ± 8	345 ± 9
PUR_BP_2	1250 ± 10	49 ± 2	322 ± 6	350 ± 7
PUR_BP_5	1550 ± 11	55 ± 1	365 ± 7	355 ± 7

**Table 3:** Selected properties of PUR composite foams with BP filler.

Sample	Average Cell Diameter (µm)	Content of Closed-Cells (%)	Apparent Density (kg m^−3^)	Thermal Conductivity (W m^−1^ K^−1^)	Contact Angle (°)	Water Uptake (%)
PUR_0	492 ± 6	90.2 ± 0.4	37.1 ± 0.7	0.026 ± 0.001	123 ± 1	20.4 ± 0.6
PUR_BP_1	450 ± 5	89.4 ± 0.3	39.2 ± 0.6	0.027 ± 0.001	120 ± 1	18.6 ± 0.5
PUR_BP_2	445 ± 5	88.1 ± 0.4	41.4 ± 0.5	0.030 ± 0.001	128 ± 1	18.9 ± 0.4
PUR_BP_5	530 ± 6	82.3 ± 0.3	44.6 ± 0.6	0.035 ± 0.001	130 ± 1	22.4 ± 0.5

**Table 4:** The results of thermogravimetric (TGA) and derivative thermogravimetry (DTG) analysis of PUR composite foams.

Sample	T_max_ (°C)			Residue at 600 °C (wt.%)
	1st Stage	2nd Stage	3rd Stage	
PUR_0	220	309	580	29.0
PUR_BP_1	224	325	586	29.1
PUR_BP_2	226	320	585	30.7
PUR_BP_5	217	302	591	31.9

**Table 5:** The results of the cone calorimeter test.

	IT (s)	pHRR (kW m^−2^)	TSR (m^2^ m^−2^)	THR (MJ m^−2^)	COY (kg kg^−1^)	CO_2_Y (kg kg^−1^)	COY/CO_2_Y (-)	LOI (%)
PUR_0	4	260	1500	21.5	0.210	0.240	0.875	20.2
PUR_BP_1	4	170	1200	20.5	0.170	0.200	0.850	20.9
PUR_BP_2	5	155	1100	20.9	0.160	0.195	0.820	21.2
PUR_BP_5	5	190	1450	21.2	0.162	0.190	0.852	20.5
